# Polymorphism rs2682818 in miR‐618 is associated with colorectal cancer susceptibility in a Han Chinese population

**DOI:** 10.1002/cam4.1409

**Published:** 2018-03-13

**Authors:** Yuetong Chen, Mulong Du, Wei Chen, Lingjun Zhu, Congye Wu, Zhengdong Zhang, Meilin Wang, Haiyan Chu, Dongying Gu, Jinfei Chen

**Affiliations:** ^1^ Department of Oncology Nanjing First Hospital Nanjing Medical University Nanjing China; ^2^ Clinical Research Center Xuyi People's Hospital Xuyi Jiangsu China; ^3^ Department of Environmental Genomics Jiangsu Key Laboratory of Cancer Biomarkers, Prevention and Treatment Collaborative Innovation Center for Cancer Personalized Medicine Nanjing Medical University Nanjing China; ^4^ Department of Genetic Toxicology The Key Laboratory of Modern Toxicology of Ministry of Education School of Public Health Nanjing Medical University Nanjing China; ^5^ Department of Biostatistics School of Public Health Nanjing Medical University Nanjing China; ^6^ Department of Digestive Disease Dongtai Hospital Affiliated to Nantong Medical University Yancheng China; ^7^ Department of Oncology The First Affiliated Hospital of Nanjing Medical University Nanjing China; ^8^ Jiangsu Key Lab of Cancer Biomarkers, Prevention and Treatment Collaborative Innovation Center for Cancer Personalized Medicine Nanjing Medical University Nanjing China

**Keywords:** Colorectal cancer, MiR‐618, SNP, susceptibility

## Abstract

MicroRNAs (miRNAs), endogenous small noncoding RNAs (ncRNAs), play crucial roles in cancer development. Many studies have demonstrated that miRNAs can serve as diagnostic and therapeutic biomarkers for malignancies. Additionally, single nucleotide polymorphisms (SNPs) located in miRNA functional regions have been reported to be involved in cancer susceptibility. In this study, we investigated the associations between SNPs located in miRNA functional regions and colorectal cancer (CRC) susceptibility. We systematically screened all candidate miRNAs and their SNPs and then evaluated the relationships between the SNPs and CRC susceptibility in a Han Chinese population including 878 patients with CRC and 884 controls. Genotyping was performed by TaqMan assay. After comprehensively screening the miRNAs and SNPs, we elected to evaluate the association between SNP rs2682818 in miR‐618 and CRC susceptibility. We found that the AA and AC/AA genotypes of rs2682818 were associated with a decreased risk of CRC compared with the CC genotype (odds ratio (OR) = 0.54, 95% confidence interval (CI) = 0.37–0.79 for AA vs. CC in codominant model; OR = 0.82, 95% CI = 0.68–0.99 for AC/AA vs. CC in dominant model). However, we obtained no statically significant results in our subgroup analyses. SNP rs2682818 in miR‐618 has potential as a biomarker for individuals with high CRC susceptibility. Our findings need to be verified in studies including larger samples. Moreover, molecular functional studies of miR‐681 must be performed to confirm its relationship with CRC.

## Introduction

Colorectal cancer (CRC) is the third most common cancer and one of the major causes of cancer‐related morbidity and mortality globally 1. The newest recommendations of the United States Preventive Services Task Force (USPSTF) state that CRC screening via colonoscopy, sigmoidoscopy, or fecal occult blood testing should start at 50 years of age and continue until 75 years of age 2. Reports from both the USA and the UK 3, 4 have verified that colonoscopy and sigmoidoscopy are significantly beneficial with respect to the prevention and early diagnosis of CRC. Fecal‐based tests also play an important role in the screening of populations with high CRC susceptibility, especially in areas lacking modern endoscopic technology 5, 6. The mechanisms underlying CRC occurrence and progression are complicated and mainly involve genetic and environmental factors, such as gender 1, heritable factors 7, fatigue 8, and physical activity 9. Various oncogenes and tumor suppressors, such as *KRAS*,* APC*, BRAF, *TP53,* and *SMAD4*, have been identified by CRC‐related studies and may be useful for diagnosing and treating CRC in the future 10, 11.

MicroRNAs (miRNAs), which are the most extensively studied category of small noncoding RNAs (ncRNAs), are 17–25 nucleotides in length 12. MiRNAs have the capability to bind to the 3′‐untranslated regions (3′‐UTRs) of messenger RNAs (mRNAs) to simultaneously suppress target gene expression and contribute to cancer susceptibility 13. MiRNAs are smaller and fewer in number than mRNAs. However, miRNAs are stable molecules capable of regulating numerous mRNAs. Thus, they can function as biomarkers and therapeutic targets 14, 15. MiRNAs regulate gene expression by several mechanisms, including the antagonization of mRNA translation. Thus, they can govern multiple biological progresses, such as cell morphogenesis, proliferation, differentiation, and apoptosis 16, 17, 18. Increasing numbers of studies have revealed that some miRNAs, such as miR‐320 and miR‐224, are related to CRC susceptibility, development, treatment, and prognoses 19, 20.

The most common forms of variation in the human genome are single nucleotide polymorphisms (SNPs), which can influence cancer susceptibility 21. SNPs within pre‐miRNAs or miRNAs can change the final levels and functions of the molecules by regulating primary transcription, pri‐miRNA and pre‐miRNA processing and maturation, and miRNA‐target interactions 22. Furthermore, some cancers, such as breast cancer 23, colorectal cancer 24, gastric cancer 25, and lung cancer 26, have been reported to be associated with miRNAs. MiRNAs undeniably play a crucial role in human cancer initiation and development 27. Thus, in this study, we aimed to verify the scientific hypothesis that some SNPs are associated with CRC susceptibility in a Han Chinese population.

## Materials and Methods

### Study participants

We enrolled a total of 878 patients with CRC and 884 healthy individuals in this study. All the patients with histopathological diagnoses of CRC were recruited from The First Affiliated Hospital and Nanjing First Hospital of Nanjing Medical University. We did not impose any restrictions regarding age or sex. Participants with a history of primary or recurrent or metastatic cancer, as well as participants who had received radiotherapy or chemotherapy, were excluded from the study. Approximately 95% of the patients who were eligible for this study were ultimately enrolled herein. The cancer‐free controls, who were genetically unrelated to the patients and had no history of cancer or suspicious clinical symptoms suggestive of CRC, were frequency matched to the patients by age (±5 years) and sex. All subjects who had smoked daily for over 1 year were considered smokers, and the remaining subjects were considered nonsmokers. All subjects who had consumed one or more glasses of alcohol weekly for at least 1 year were considered drinkers, and the remaining subjects were considered nondrinkers. Trained researchers interviewed all the subjects face to face using a guided questionnaire covering demographic factors and life exposures. The overall response rate of the subjects enrolled in the study was >85%. After signing the informed consent and providing the above information, each participant provided a 5‐mL peripheral venous blood sample for genomic DNA extraction.

### SNP screening

The following databases were integrated to screen candidate SNPs: miRBase (http://microrna.sanger.ac.uk/, version 10.0), dbSNP (http://ncbi.nlm.nih.gov/SNP), HapMap (http://www.hapmap.org), and Patrocles (http://www.patrocles.org/). An algorithm input into miRanda was used to evaluate the effects of SNPs on miRNA structural folding.

### SNP genotyping

TaqMan assay was implemented to genotype genomic DNA obtained from the above‐mentioned whole‐blood samples. The sequences of the primers and probes specific for each candidate SNP can be provided if requested. We used a 384‐well ABI 7900HT Real‐time PCR System (Applied Biosystems, Foster City, CA) to amplify the genomes of all the samples. We utilized SDS 2.4 software (Applied Biosystems) to analyze allelic discrimination. Two researchers independently performed the genotype assay in a blinded manner. Furthermore, our laboratory technicians randomly selected and analyzed approximately 10% of the samples to confirm their quality. The concordance rate reached 100%.

### Statistical analysis

The Hardy–Weinberg equilibrium (HWE) of the candidate SNPs in control samples was calculated by a goodness‐of‐fit chi‐square test. We calculated adjusted odds ratios (ORs) and 95% confidence intervals (CIs) to evaluate the relationships between the candidate SNPs and CRC susceptibility using multivariate unconditional logistic regression models, namely, dominant, recessive, codominant, and additive models. *P *<* *0.05 was considered statistically significant. All the data were two‐sided and were jointly analyzed by two researchers using SAS soft (version 9.1.3; SAS Institute Inc, Cary, NC).

## Results

### Characteristics of the study population

The characteristics of the 878 patients with CRC and the 884 cancer‐free controls are shown in Table 1. There were no significant differences in age (*P *=* *0.632), gender (*P *=* *0.125), smoking status (*P *=* *0.187), or drinking status (*P *=* *0.222) between the patients and controls. However, the patients with CRC were more likely to have a family history of cancer (23.5%) than the control subjects (9.8%; *P *<* *0.001). Additionally, 52.3% of the patients were suffering from colon cancer, and the remaining patients were suffering from rectal cancer (47.7%). We further categorized the patients according to the tumor grade (low, 7.1%; intermediate, 77.1%; high, 15.8%) and Dukes stage (A, 7.0%; B, 44.6%; C, 36.6%; D, 11.8%).

**Table 1:** Characteristics of the patients with CRC and the controls

Variables	Cases (*n* = 878)		Controls (*n* = 884)		*P‐*value
	*n*	%	*n*	%	
Age (mean ± SD)	60.0 ± 12.9		60.3 ± 13.7		0.632
Gender					
Male	541	61.6	513	58	0.125
Female	337	38.4	371	42	
Smoking status					
None	580	66.1	610	69	0.187
Smoker	298	33.9	274	31	
Drinking status					
None	636	72.4	663	75	0.222
Drinker	242	27.6	221	25	
Family history of cancer					
No	672	76.5	797	90.2	<0.001
Yes	206	23.5	87	9.8	
Tumor site					
Colon	459	52.3			
Rectum	419	47.7			
Tumor grade					
Low	62	7.1			
Intermediate	677	77.1			
High	139	15.8			
Stage					
A	61	7.0			
B	392	44.6			
C	321	36.6			
D	104	11.8			

### Screening for candidate SNPs

**Figure 1:**
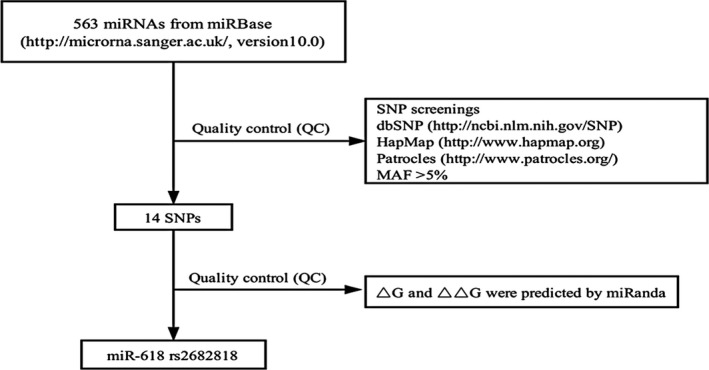
Systematic SNP screening strategy.

**Figure 2:**
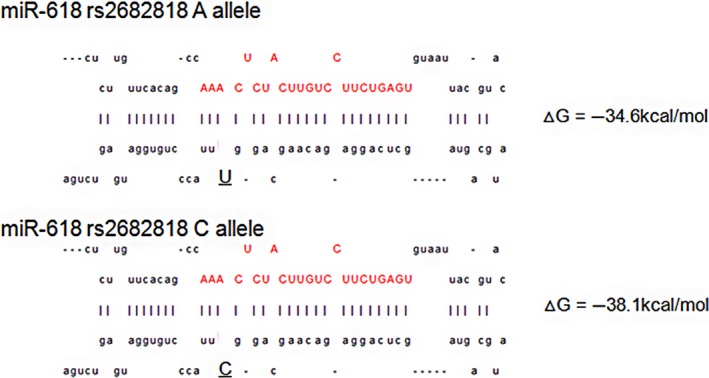
In silico prediction of the ▵G for miR‐618 rs2682818.

We systematically screened for SNPs (Fig. 1). SNPs located in miRNA functional regions were captured by the miRBase and dbSNP databases. The Han Chinese population (CHB) of HapMap was used as a reference population to determine the frequencies of the candidate SNPs, and Patrocles software was used to determine the potential function of every SNP. Through these analyses, we selected a total of 14 SNPs with a minor allele frequency (MAF) >0.05 (Table 2). The binding energy of the hydrogen bond and the change in the structural folding energy of each candidate pre‐miRNA SNP allele (▵G) were subsequently predicted by a miRanda algorithm. SNP rs2682818 C>A in miR‐618 was found to have the greatest ▵▵G (3.50 kcal/mol, Table 2 and Fig. 2). Hence, the subsequent case–control study focused on this SNP.

**Table 2:** Basic information pertaining to the SNPs of the 14 candidate miRNAs

MiRNA	Mature miRNA sequence	SNP	Base	MAF*	▵G	▵▵G
miR‐27a	UUCACAGUGGCUAAGUUCCGC	rs895819	C>T	0.311	−39.4/−39.4	0.00
miR‐146a	UGAGAACUGAAUUCCAUGGGUU	rs2910164	G>C	0.444	−42.4/−39.6	2.80
miR‐149	UCUGGCUCCGUGUCUUCACUCCC	rs2292832	T>C	0.267	−52.7/−54.9	2.20
miR‐196a2	UAGGUAGUUUCAUGUUGUUGGG	rs11614913	T>C	0.489	−44.5/−47.1	2.60
miR‐423	UGAGGGGCAGAGAGCGAGACUUU	rs6505162	C>A	0.2	−48.8/−48.8	0.00
miR‐492	AGGACCUGCGGGACAAGAUUCUU	rs2289030	C>G	0.239	−39.0/−40.7	1.70
miR‐499	UUAAGACUUGCAGUGAUGUUU	rs3746444	A>G	0.174	−61.9/−62.3	0.40
miR‐603	CACACACUGCAAUUACUUUUGC	rs11014002	C>T	0.25	−40.5/−42.3	1.80
miR‐604	AGGCUGCGGAAUUCAGGAC	rs2368392	C>T	0.262	−27.3/−26.7	0.60
miR‐605	UAAAUCCCAUGGUGCCUUCUCCU	rs2043556	A>G	0.344	−52.3/−54.9	2.60
miR‐608	AGGGGUGGUGUUGGGACAGCUCCGU	rs4919510	C>G	0.433	−31.7/−30.9	0.80
**miR‐618**	**AAACUCUACUUGUCCUUCUGAGU**	**rs2682818**	**C>A**	**0.32**	−**38.1/**−**34.6**	**3.50**
miR‐923	GUCAGCGGAGGAAAAGAAACU	rs4796042	G>C	0.314	−13.0/−9.6	3.40
miR943	CUGACUGUUGCCGUCCUCCAG	rs1077020	T>C	0.256	−43.3/−42.2	1.10

*MAF, minor allele frequency.

### Evaluation of the association between rs2682818 and CRC risk

The results of the logistic regression analysis of the distributions of the genotypes and alleles of the miR‐618 rs2682818 polymorphism in the patient and control groups are summarized in Table 3. The distributions of the miR‐618 rs2682818 genotypes in the control group were found to be in HWE (*P *=* *0.457). The frequencies of the rs2682818 genotypes were 54.1% (CC), 40.2% (AC), and 5.7% (AA) in the patient group and were significantly different from those in the control group (49.3% (CC), 41.1% (AC), and 9.6% (AA)) (*P *=* *0.004). Individuals carrying the AA or AC/AA genotype had a lower CRC susceptibility than individuals carrying the CC genotype (OR = 0.54, 95% CI = 0.37–0.79 for AA vs. CC in codominant model; OR = 0.82, 95% CI = 0.68–0.99 for AC/AA vs. CC in dominant model, Table 3).

**Table 3:** MiR‐618 rs2682818 genotype frequencies and distributions

Genotype	Cases (*n* = 878)		Controls (*n* = 884)		*P‐*value^a^	OR (95% CI)^a^
	*N*	%	*N*	%		
CC	475	54.1	436	49.3		1.00
AC	353	40.2	363	41.1	0.241	0.89 (0.73–1.08)
AA	50	5.7	85	9.6	0.001	0.54 (0.37–0.79)
A allele	0.258		0.302		0.004	
AC/AA	403	45.9	448	50.7	0.042	0.82 (0.68–0.99)

a Odds ratios were adjusted for age, gender, and smoking status. CI, confidence interval.

### Subgroup analysis of the relationship between rs2682818 and CRC risk

We evaluated the relationship between rs2682818 and CRC susceptibility in subgroups of patients stratified according to their demographic features and clinical characteristics. Unfortunately, we did not observe a significant association between rs2682818 and CRC susceptibility in either subgroup (Tables S1 and S2).

## Discussion

The principle objective of this study was to determine the association between a miR‐618 polymorphism and CRC susceptibility in a Han Chinese population. A lower susceptibility to CRC was noted among specimens with an rs2682818 AA or AC/AA genotype than among specimens with a CC genotype. However, we did not observe any statically significant associations in the subsequent subgroup analyses.

Previous studies have reported that miR‐618 is associated with malignancies, including hepatocellular tumors 28, breast cancer 29, Barrett's esophageal cancer 30, and lymphadenoma 31, indicating that miR‐618 may be useful as a cancer biomarker or therapeutic target. One previous study 31 showed that SNP rs2682818, which is located in the hairpin‐loop structure of the miR‐618 precursor, can serve as a risk biomarker for and therapeutic target in follicular lymphoma, as miR‐618 regulates lymphomagenic pathways. However, thus far, the role of miR‐618 in CRC susceptibility is unclear. Thus, we evaluated the association between rs2682818 in miR‐618 and CRC susceptibility in a case–control study involving a Han Chinese population.

MiR‐618 gradually accumulates in hormone‐stimulated cells, and the expression levels of its targets are clearly enriched in MCF‐7 cells 32. Low‐density lipoprotein receptor‐related protein 12 (LPR12) has been identified as one putative target of miR‐618 and is expressed at lower levels in malignant tissues than in adjacent normal tissues 33. Thus, enhancements of miR‐618 expression may reduce LPR12 levels and accelerate hepatocellular carcinoma (HCC) development. One study 28 suggested that increased miR‐618 expression was a biomarker for HCC, especially among hepatitis C virus (HCV) carriers.

Fu et al. 31 investigated the link between SNP rs2682818 in miR‐618 and follicular lymphoma and demonstrated that the formation of the pri‐miR‐618 stem‐loop and/or the process by which the pri‐ or pre‐miR‐618 stem‐loop interacts with its target can be disrupted by a change in the variant T allele. The authors ultimately found that mature miR‐618 expression but not pri‐miR‐618 expression was decreased in cells transfected with the variant precursor compared with cells transfected with the wild‐type precursor. This valuable study showed that the process by which miR‐618 interacts with its target can be disrupted by a functional rs2682818 in the stem‐loop sequence of its precursor. This phenomenon may affect several downstream pathways.

Several limitations should be considered when interpreting our results. First, information regarding some exposure variables, such as dietary preferences, mental status, and chronic disease histories, was not easy to acquire via the restricted questionnaire interviews. Second, the sample sizes of this case–control study were relatively insufficient, and the selection of only one central Han Chinese population adversely affected the representativeness of our results and may render our results less powerful than those of similar studies. The above limitations may explain why we noted no statistically significant results in the subgroup analyses in which the patients were stratified according to their demographic and clinical characteristics. In addition, SNP rs2682818 C>A in miR‐618, whose ▵▵G (3.50 kcal/mol) was the greatest among all 14 candidates analyzed herein, was the only candidate SNP that was analyzed extensively in our study.

In conclusion, our study revealed that polymorphism rs2682818 in miR‐618 contributes to CRC susceptibility. As this conclusion is based merely on our analysis of a Han Chinese population of limited size, our findings should be validated in studies including larger samples. Moreover, functional studies of miR‐618 are needed to confirm its relationship with CRC in the future.

## Ethics Statement

The Institutional Review Board of Nanjing Medical University ratified this study before we began our experiments. Every single participant signed an informed consent, and the research protocol complied fully with approved guidelines.

## Conflict of Interest

The authors have no disclosures.

## Supporting information


**Table S1.** MiR‐618 rs2682818 genotype frequencies and distributions according to demographic characteristics.
**Table S2.** MiR‐618 rs2682818 genotype frequencies and distributions according to patient clinical characteristics.
