# Supplementary material for: Polymorphism rs2682818 in miR‐618 is associated with colorectal cancer susceptibility in a Han Chinese population
Source: Cancer Med. 2018 Mar 13;7(4):1194–200. doi: 10.1002/cam4.1409 (PMC5911622; doi:10.1002/cam4.1409)
Supplement: Supplementary file 1 — Table S1. MiR‐618 rs2682818 genotype frequencies and distributions according to demographic characteristics. Table S2. MiR‐618 rs2682818 genotype frequencies and distributions according to patient clinical characteristics. [file CAM4-7-1194-s001.docx]

**Supplementary Table 1.** MiR-618 rs2682818 genotype frequencies and distributions according to demographic characteristics

| **Variables** | **Total** | **CC** |  | **AC/AA** |  | **AC/AA vs. CC** | | |
| --- | --- | --- | --- | --- | --- | --- | --- | --- |
|  | **N (case/control)** | **N (case/control)** |  | **N (case/control)** |  | ***P* value^a^** | | **OR (95% CI)^a^** |
| Age (years) |  |  |  |  |  |  |  | |
| ≤60 | 440/401 | 238/200 |  | 202/201 |  | 0.234 | 0.85 (0.64-1.11) | |
| >60 | 438/483 | 237/236 |  | 201/247 |  | 0.076 | 0.79 (0.61-1.03) | |
| Gender |  |  |  |  |  |  |  | |
| Male | 541/513 | 287/250 |  | 254/263 |  | 0.164 | 0.84 (0.66-1.07) | |
| Female | 337/371 | 188/186 |  | 149/185 |  | 0.148 | 0.80 (0.59-1.08) | |
| Smoking status |  |  |  |  |  |  |  | |
| Smoker | 298/274 | 158/137 |  | 140/137 |  | 0.420 | 0.87 (0.63-1.22) | |
| None | 580/610 | 317/299 |  | 263/311 |  | 0.052 | 0.79 (0.63-1.01) | |
| Drinking status |  |  |  |  |  |  |  | |
| Drinker | 242/221 | 123/112 |  | 119/109 |  | 0.729 | 0.94 (0.64-1.36) | |
| None | 636/663 | 352/324 |  | 284/339 |  | 0.055 | 0.76 (0.61-1.03) | |
| Family history of cancer | |  |  |  |  |  |  | |
| Yes | 206/87 | 114/46 |  | 92/41 |  | 0.764 | 0.93 (0.56-1.54) | |
| No | 672/797 | 361/390 |  | 311/407 |  | 0.064 | 0.82 (0.67-1.01) | |
| ^a^Odds ratios were adjusted for age, gender and smoking status. CI, confidence interval. | | | | | | | | |

**Supplementary Table 2.** MiR-618 rs2682818 genotype frequencies and distributions according to patient clinical characteristics

| **Variables** | **CC** | |  | **CA/AA** | |  | **CA/AA vs. CC** | |
| --- | --- | --- | --- | --- | --- | --- | --- | --- |
|  | **N** | **%** |  | **N** | **%** |  | ***P* value^a^** | **OR (95% CI)^a^** |
| Stage |  |  |  |  |  |  |  |  |
| A/B | 247 | 54.5 |  | 206 | 45.5 |  |  | 1.00 |
| C/D | 228 | 53.7 |  | 197 | 46.3 |  | 0.880 | 0.98 (0.75-1.28) |
| Grade |  |  |  |  |  |  |  |  |
| Poor/Moderate | 396 | 53.6 |  | 343 | 46.4 |  |  | 1.00 |
| Well | 79 | 56.8 |  | 60 | 43.2 |  | 0.412 | 1.17 (0.81-1.69) |
| Tumor site |  |  |  |  |  |  |  |  |
| Colon | 251 | 54.7 |  | 208 | 45.3 |  |  | 1.00 |
| Rectum | 224 | 53.5 |  | 195 | 46.5 |  | 0.717 | 0.95 (0.73-1.24) |

^a^ Odds ratios were adjusted for age, gender and smoking status. CI, confidence interval.
